# Supplementary material for: Immunotherapy-related cognitive impairment after CAR T cell therapy in mice
Source: bioRxiv. 2024 May 14:2024.05.14.594163. Preprint. [Version 1] doi: 10.1101/2024.05.14.594163 (PMC11118392; doi:10.1101/2024.05.14.594163)
Supplement: Supplement 1 [file NIHPP2024.05.14.594163v1-supplement-1.pdf]

bioRxiv preprint doi: https://doi.org/10.1101/2024.05.14.594163; this version posted May 14, 2024. The copyright holder for this preprint (which was not certified by peer review) is the author/funder, who has granted bioRxiv a license to display the preprint in perpetuity. It is made available under aCC-BY 4.0 International license.

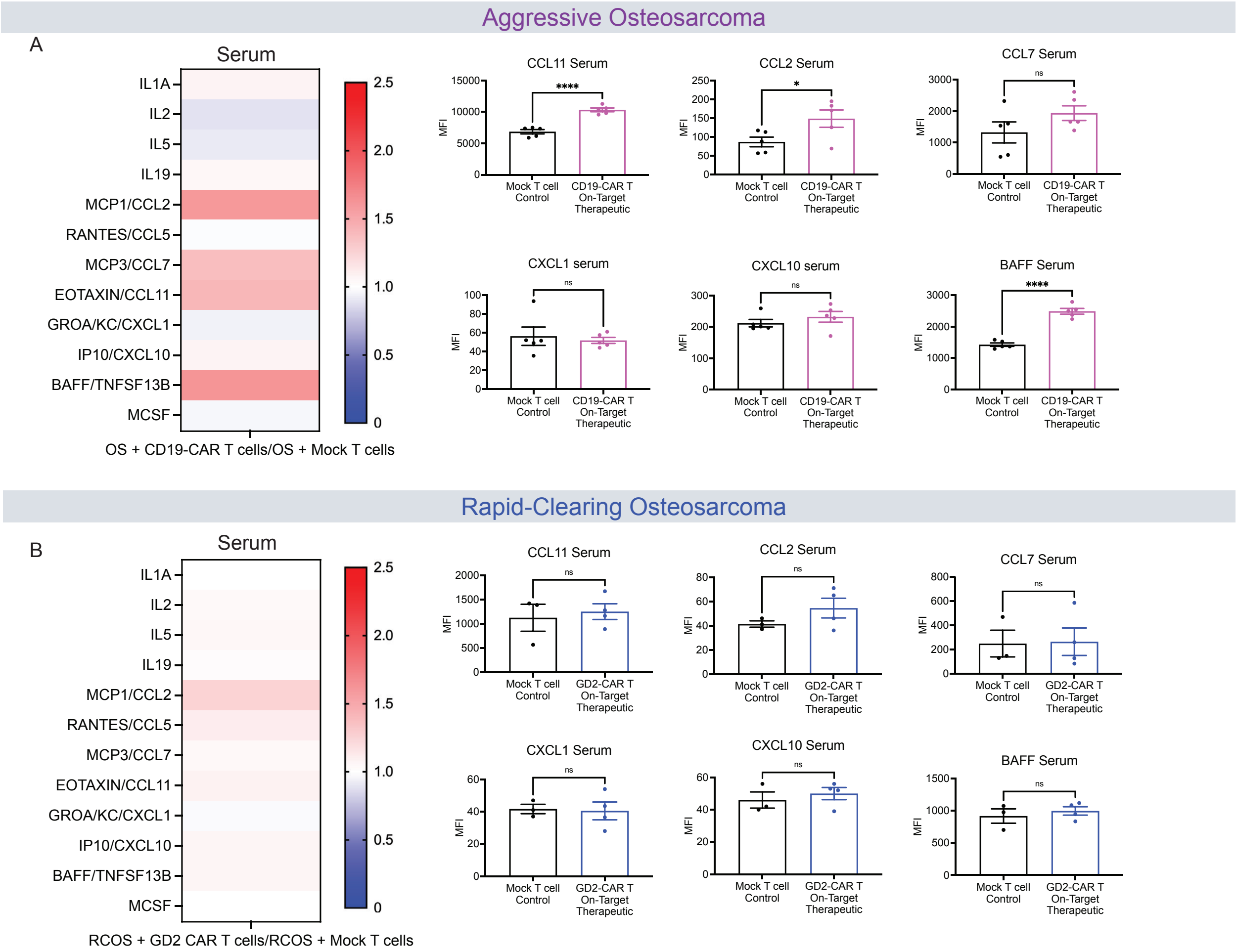


**Supplemental Figure 1: Cytokine/chemokine measurements in the serum in two Osteosarcoma models**

(A) Left, heat map of fold change of cytokine and chemokine analysis of serum in aggressive osteosarcoma (OS)+On-Target Therapeutic CD19-CAR T cell treated mice relative to aggressive osteosarcoma (OS)+mock T cell treated control mice 35-days post-CAR T cell therapy. Right, quantification of raw MFI of serum levels of cytokines (CCL11, CCL2, CCL7, CXCL1, CXCL10, BAFF).

(B) Left, heat map of fold change of cytokine and chemokine analysis of serum in rapid-clearing osteosarcoma (RCOS)+On-Target Therapeutic GD2-CAR T cell treated mice relative to rapid-clearing osteosarcoma (RCOS)+mock T cell treated control mice 35-days post-CAR T cell therapy. Right, quantification of raw MFI of serum levels of cytokines (CCL11, CCL2, CCL7, CXCL1, CXCL10, BAFF).

(A and B) Cytokine and chemokines significantly elevated or depressed, analyzed via Student's T test of raw MFI values.

bioRxiv preprint doi: https://doi.org/10.1101/2024.05.14.594163; this version posted May 14, 2024. The copyright holder for this preprint (which was not certified by peer review) is the author/funder, who has granted bioRxiv a license to display the preprint in perpetuity. It is made available under aCC-BY 4.0 International license.

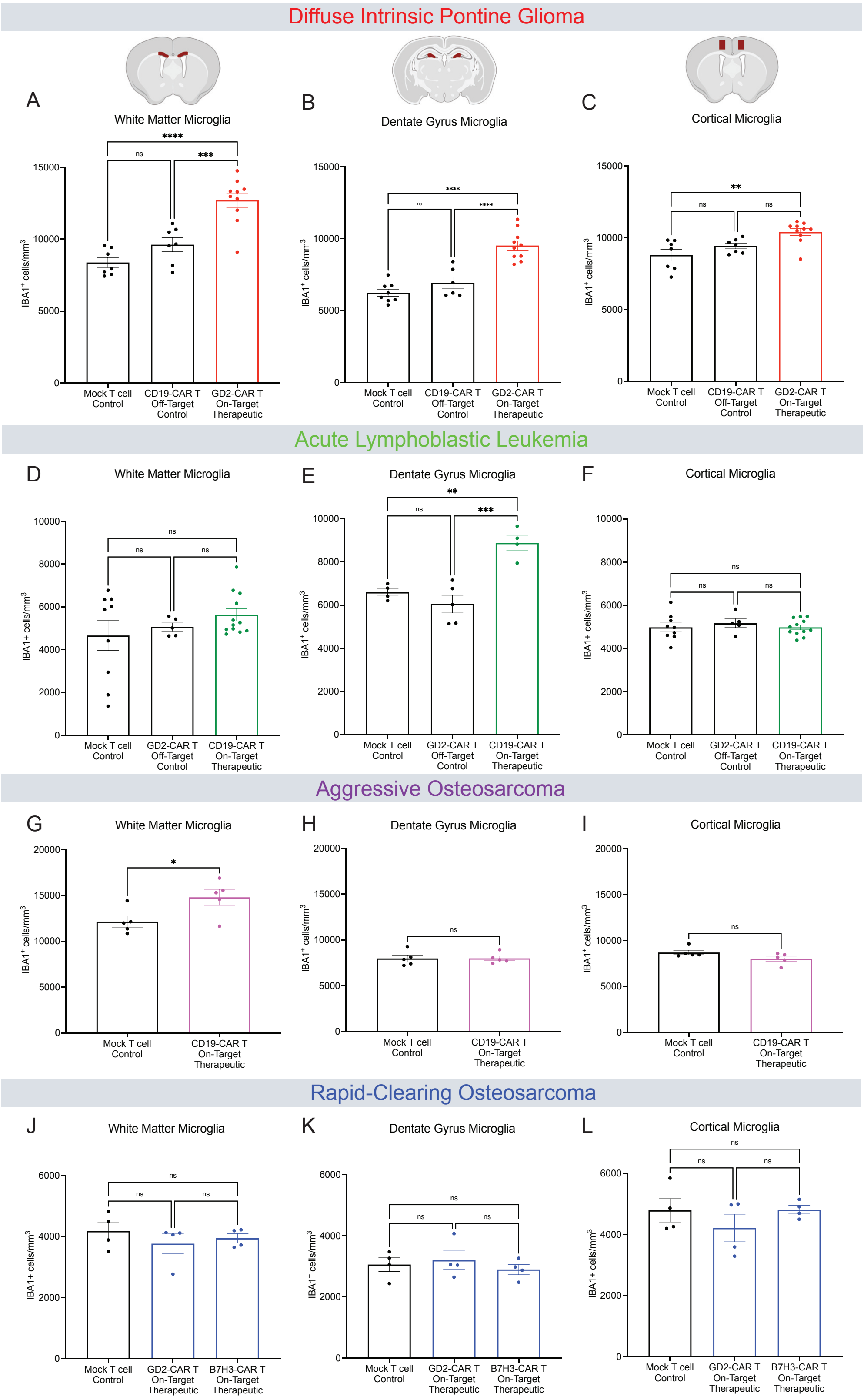


**Supplemental Figure 2. Microglial density in the white matter, dentate gyrus, and cortex**

bioRxiv preprint doi: https://doi.org/10.1101/2024.05.14.594163; this version posted May 14, 2024. The copyright holder for this preprint (which was not certified by peer review) is the author/funder, who has granted bioRxiv a license to display the preprint in perpetuity. It is made available under aCC-BY 4.0 International license.

(A) Microglia (IBA1+) quantification in the corpus callosum white matter 35 days post-CAR T therapy in the DIPG model. Mock T cell Control (n=7 mice), CD19-CAR T Control (n=7 mice), GD2-CAR T Therapeutic (n=10 mice).
(B) Microglia (IBA1+) quantification in the hilar white matter region of the dentate gyrus 35 days post-CAR T therapy in the DIPG model. Mock T cell Control (n=8 mice), CD19-CAR T Control (n=6 mice), GD2-CAR T Therapeutic (n=10 mice).
(C) Microglia (IBA1+) quantification in the cortical gray matter 35 days post-CAR T therapy in the DIPG model. Mock T cell Control (n=7 mice), CD19-CAR T Control (n=7 mice), GD2-CAR T Therapeutic (n=10 mice).
(D) Microglia (IBA1+) quantification in the corpus callosum white matter 35 days post-CAR T therapy in the ALL model. Mock T cell Control (n=9 mice), GD2-CAR T Control (n=5 mice), CD19-CAR T Therapeutic (n=12 mice).
(E) Microglia (IBA1+) quantification in the hilar white matter region of the dentate gyrus 35 days post-CAR T therapy in the ALL model. Mock T cell Control (n=4 mice), GD2-CAR T Control (n=5 mice), CD19-CAR T Therapeutic (n=4 mice).
(F) Microglia (IBA1+) quantification in the cortical gray matter 35 days post-CAR T therapy in the ALL model. Mock T cell Control (n=9 mice), GD2-CAR T Control (n=5 mice), CD19-CAR T Therapeutic (n=12 mice).
(G) Microglia (IBA1+) quantification in the corpus callosum white matter 35 days post-CAR T therapy in the aggressive osteosarcoma model. Mock T cell Control (n=5 mice), CD19-CAR T Therapeutic (n=5 mice).
(H) Microglia (IBA1+) quantification in the hilar white matter region of the dentate gyrus 35 days post-CAR T therapy in the aggressive osteosarcoma model. Mock T cell Control (n=5 mice), CD19-CAR T Therapeutic (n=5 mice).
(I) Microglia (IBA1+) quantification in the cortical gray matter 35 days post-CAR T therapy in the aggressive osteosarcoma model. Mock T cell Control (n=5 mice), CD19-CAR T Therapeutic (n=5 mice).
(J) Microglia (IBA1+) quantification in the corpus callosum white matter 35 days post-CAR T therapy in the rapid-clearing osteosarcoma model. Mock T cell Control (n=4 mice), GD2-CAR T Therapeutic (n=4 mice), B7H3-CAR T Therapeutic (n=4 mice).
(K) Microglia (IBA1+) quantification in the hilar white matter region of the dentate gyrus 35 days post-CAR T therapy in the rapid-clearing osteosarcoma model. Mock T cell Control (n=4 mice), GD2-CAR T Therapeutic (n=4 mice), B7H3-CAR T Therapeutic (n=4 mice).
(L) Microglia (IBA1+) quantification in the cortical gray matter 35 days post-CAR T therapy in the rapid-clearing osteosarcoma model. Mock T cell Control (n=4 mice), GD2-CAR T Therapeutic (n=4 mice), B7H3-CAR T Therapeutic (n=4 mice).

Data shown as mean ± SEM (A-F, J-L). Each point = one mouse. ns = $p > 0.05$, *$p < 0.05$, **$p < 0.01$, ***$p < 0.001$, ****$p < 0.0001$, analyzed via One-way ANOVA.
(G-I) Data shown as mean ± SEM. Each point = one mouse, ns = $p > 0.05$, *$p < 0.05$, analyzed via Unpaired T Test.

bioRxiv preprint doi: https://doi.org/10.1101/2024.05.14.594163; this version posted May 14, 2024. The copyright holder for this preprint (which was not certified by peer review) is the author/funder, who has granted bioRxiv a license to display the preprint in perpetuity. It is made available under aCC-BY 4.0 International license.

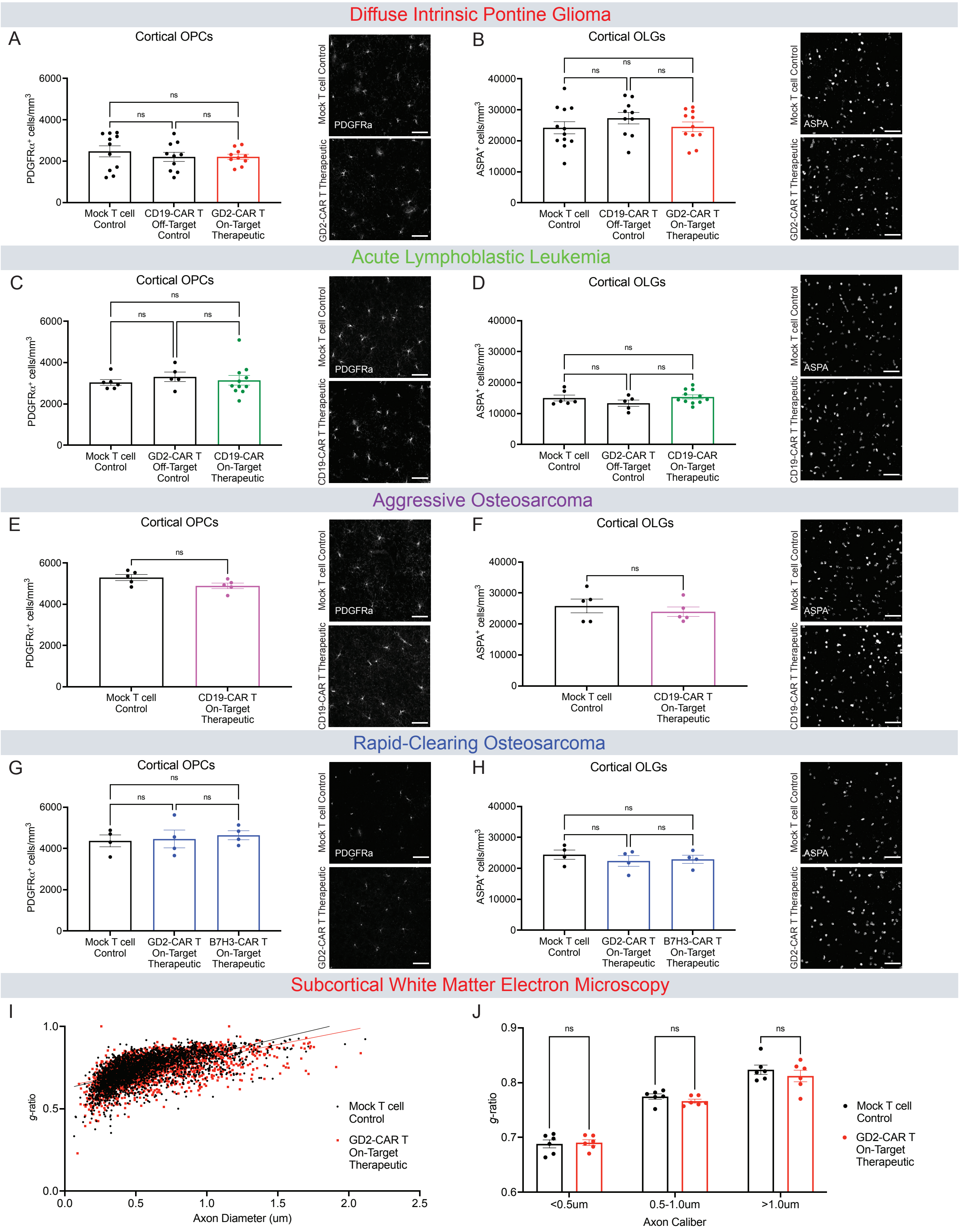


**Supplemental Figure 3. Normal cortical oligodendroglia and subcortical myelin sheath thickness following tumor-clearing CAR T cell therapy**

bioRxiv preprint doi: https://doi.org/10.1101/2024.05.14.594163; this version posted May 14, 2024. The copyright holder for this preprint (which was not certified by peer review) is the author/funder, who has granted bioRxiv a license to display the preprint in perpetuity. It is made available under aCC-BY 4.0 International license.

(A) Quantification and representative confocal micrographs of PDGFRa+ oligodendrocyte precursor cells from the cortical gray matter in DIPG xenografted mice 35 days post-CAR T therapy. Mock T cell Control (n=11 mice), CD19-CAR T Control (n=10 mice), GD2-CAR T Therapeutic (n=10 mice).
(B) Quantification and representative confocal micrographs of ASPA+ mature oligodendrocytes from the cortical gray matter in DIPG xenografted mice 35 days post-CAR T therapy. Mock T cell Control (n=12 mice), CD19-CAR T Control (n=10 mice), GD2-CAR T Therapeutic (n=11 mice).
(C) Quantification and representative confocal micrographs of PDGFRa+ oligodendrocyte precursor cells from the cortical gray matter in ALL xenografted mice 28 days post-CAR T therapy. Mock T cell Control (n=6 mice), GD2-CAR T Control (n=5 mice), CD19-CAR T Therapeutic (n=11 mice).
(D) Quantification and representative confocal micrographs of ASPA+ mature oligodendrocytes from the cortical gray matter in ALL xenografted mice 28 days post-CAR T therapy. Mock T cell Control (n=6 mice), CD19-CAR T Control (n=5 mice), GD2-CAR T Therapeutic (n=10 mice).
(E) Quantification and representative confocal micrographs of PDGFRa+ oligodendrocyte precursor cells from the cortical gray matter in aggressive osteosarcoma xenografted mice 35 days post-CAR T therapy. Mock T cell Control (n=5 mice), CD19-CAR T Therapeutic (n=5 mice).
(F) Quantification and representative confocal micrographs of ASPA+ mature oligodendrocytes from the cortical gray matter in aggressive osteosarcoma xenografted mice 35 days post-CAR T therapy. Mock T cell Control (n=5 mice), CD19-CAR T Therapeutic (n=5 mice).
(G) Quantification and representative confocal micrographs of PDGFRa+ oligodendrocyte precursor cells from the cortical gray matter in rapid-clearing osteosarcoma xenografted mice 35 days post-CAR T therapy. Mock T cell Control (n=4 mice), GD2-CAR T Therapeutic I (n=4 mice), B7H3-CAR T Therapeutic (n=4 mice).
(H) Quantification and representative confocal micrographs of ASPA+ mature oligodendrocytes from the cortical gray matter in rapid-clearing osteosarcoma xenografted mice 35 days post-CAR T therapy. Mock T cell Control (n=4 mice), GD2-CAR T Therapeutic I (n=4 mice), B7H3-CAR T Therapeutic (n=4 mice).
(I) Scatterplots of g-ratio relative to axon diameter at 35 days post-CAR T cell therapy for mock T cell control (black points) or GD2-CAR T On-Target Therapeutic (red points) treated mice. (n=6 mice per group)
(J) G-ratio relative to small (<0.5um), medium (0.5-1.0um), and large (>1.0um) caliber axons for mock T cell control (black points) or GD2-CAR T On-Target Therapeutic (red points) treated mice. (n=6 mice per group)

(A-D, G-H, J) Data shown as mean ± SEM. Each point = one mouse. ns = $p > 0.05$, analyzed via One-way ANOVA.
(E & F) Data shown as mean ± SEM. Each point = one mouse, ns = $p > 0.05$, analyzed via Unpaired T Test. Scale bars equal 50um in all confocal images.

bioRxiv preprint doi: https://doi.org/10.1101/2024.05.14.594163; this version posted May 14, 2024. The copyright holder for this preprint (which was not certified by peer review) is the author/funder, who has granted bioRxiv a license to display the preprint in perpetuity. It is made available under aCC-BY 4.0 International license.

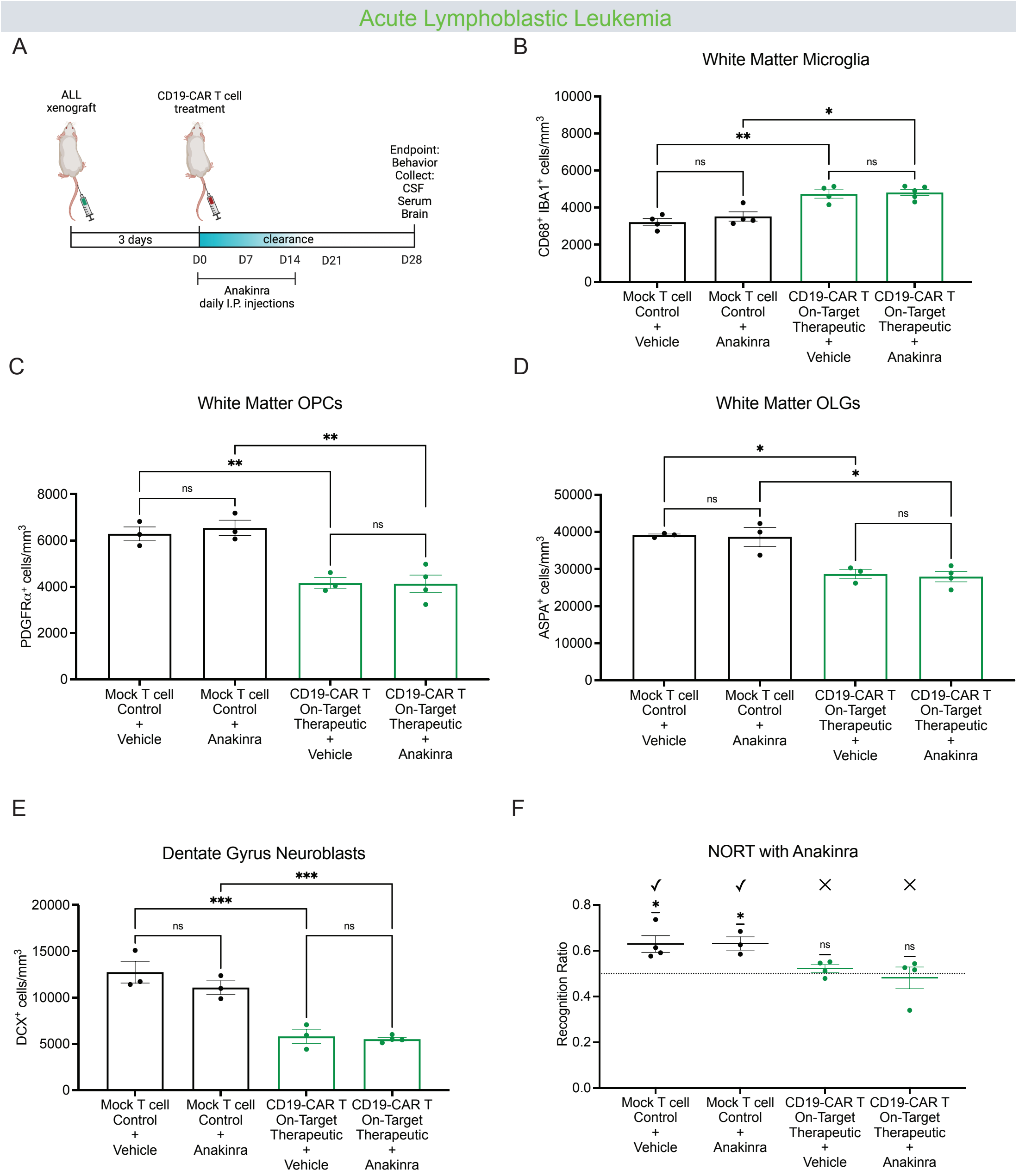


**Supplemental Figure 4. Anakinra does not rescue cellular or behavioral deficits**

(A) Schematic illustration denoting the timeline for the ALL model with Anakinra administration. (B) Quantification of corpus callosum reactive white matter microglia (IBA1+ CD68+) 28 days post-CAR T therapy with or without 14 days of Anakinra administration in ALL-xenografted mice. Mock T cell+Vehicle (n=4 mice), mock T cell+Anakinra (n=4 mice), CD19-CAR T Therapeutic+Vehicle (n=4 mice), CD19-CAR T Therapeutic+Anakinra (n=5 mice). (C) Quantification of PDGFRa+ oligodendrocyte precursor cells from the corpus callosum in ALL-xenografted mice 28 days post-CAR T therapy with or without 14 days of Anakinra administration. Mock T cell+Vehicle (n=4 mice), mock T cell+Anakinra (n=4 mice), CD19-CAR T Therapeutic+Vehicle (n=4 mice), CD19-CAR T Therapeutic+Anakinra (n=5 mice). (D) Quantification of ASPA+ mature oligodendrocytes from the corpus callosum in ALL-xenografted mice 28 days 28 days post-CAR T therapy with or without 14 days of Anakinra

bioRxiv preprint doi: https://doi.org/10.1101/2024.05.14.594163; this version posted May 14, 2024. The copyright holder for this preprint (which was not certified by peer review) is the author/funder, who has granted bioRxiv a license to display the preprint in perpetuity. It is made available under aCC-BY 4.0 International license.

administration. Mock T cell+Vehicle (n=4 mice), mock T cell+Anakinra (n=4 mice), CD19-CAR T Therapeutic+Vehicle (n=4 mice), CD19-CAR T Therapeutic+Anakinra (n=5 mice).
(E) Quantification of dentate gyrus neuroblasts (DCX+) 28 days post-CAR T therapy with or without 14 days of Anakinra administration in ALL-xenografted mice. Mock T cell+Vehicle (n=3 mice), mock T cell+Anakinra (n=3 mice), CD19-CAR T Therapeutic+Vehicle (n=3 mice), CD19-CAR T Therapeutic+Anakinra (n=4 mice).
(F) NORT performed for ALL-xenografted mice analyzed on day 28 post-CAR T therapy with or without Anakinra administration. Mock T cell+Vehicle (n=4 mice), mock T cell+Anakinra (n=3 mice), CD19-CAR T Therapeutic+Vehicle (n=4 mice), CD19-CAR T Therapeutic+Anakinra (n=4 mice).

(B, C, D, E) Data shown as mean ± SEM. Each point = one mouse. ns = $p > 0.05$, **$p < 0.01$, ***$p < 0.001$, analyzed via 2-way ANOVA.
(F) Data shown as mean ± SEM. Each point = one mouse. ns = $p > 0.05$, *$p < 0.05$, **$p < 0.01$, ****$p < 0.0001$. Analyzed via One Sample T and Wilcoxon Test.

bioRxiv preprint doi: https://doi.org/10.1101/2024.05.14.594163; this version posted May 14, 2024. The copyright holder for this preprint (which was not certified by peer review) is the author/funder, who has granted bioRxiv a license to display the preprint in perpetuity. It is made available under aCC-BY 4.0 International license.

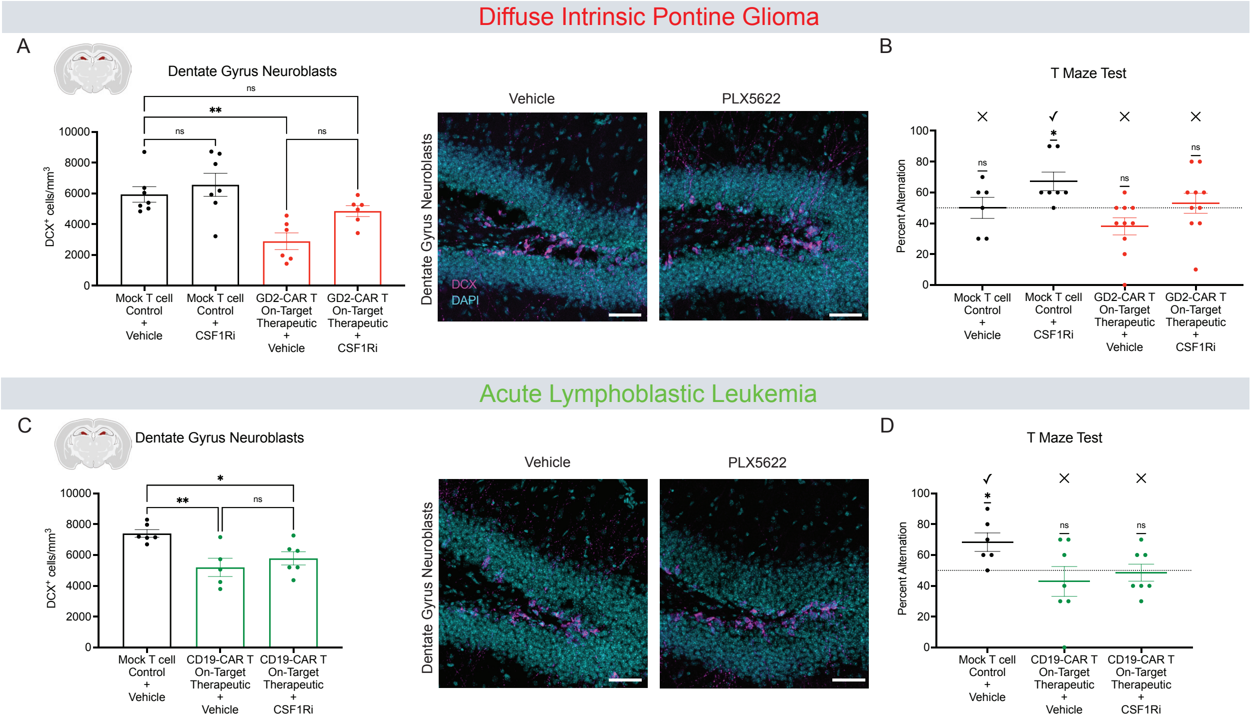


**Supplemental Figure 5. Incomplete microglial depletion in the hippocampus does not rescue behavioral deficits**

(A) Quantification of dentate gyrus neuroblasts (DCX+) 35 days post-CAR T therapy with or without 14 days of CSF1R inhibition in DIPG-xenografted mice,. Mock T cell+Vehicle Chow (n=7 mice), mock T cell+CSF1Ri Chow (n=7 mice), GD2-CAR T Therapeutic+Vehicle Chow (n=6 mice), GD2-CAR T Therapeutic+CSF1Ri Chow (n=6 mice). Representative confocal micrographs of neuroblasts (magenta = DCX, cyan = DAPI) in the dentate gyrus of DIPG xenografted mice 35 days after CAR T cell therapy with or without 14 days of CSF1R inhibitor (PLX5622).

(B) T Maze Test results from DIPG-xenografted mice D35 post-CAR T therapy with or without 14 days of CSF1R inhibition. Mock T cell+Vehicle Chow (n=6 mice), mock T cell+CSF1Ri Chow (n=7 mice), GD2-CAR T Therapeutic+Vehicle Chow (n=10 mice), GD2-CAR T Therapeutic+CSF1Ri Chow (n=10 mice).

(C) Quantification of dentate gyrus neuroblasts (DCX+) in ALL-xenografted mice at 28 days post-CAR T therapy with or without 14 days of CSF1R inhibition. Mock T cell+Vehicle Chow (n=6 mice), CD19-CAR T Therapeutic+Vehicle Chow (n=5 mice), CD19-CAR T Therapeutic+CSF1Ri Chow (n=6 mice). Representative confocal micrographs of neuroblasts (magenta = DCX, cyan = DAPI) in the dentate gyrus of ALL xenografted mice 28 days after CAR T cell therapy with or without 14 days of CSF1R inhibitor (PLX5622).

(D) T maze Test results from ALL-xenografted mice at D28 post-CAR T therapy with or without 14 days of CSF1R inhibition. Mock T cell+Vehicle Chow (n=6 mice), CD19-CAR T Therapeutic+Vehicle Chow (n=7 mice), CD19-CAR T Therapeutic+CSF1Ri Chow (n=7 mice).

Data shown as mean ± SEM (A-D). Each point = one mouse. ns = $p > 0.05$, * $p < 0.05$, ** $p < 0.01$, analyzed via 2-way ANOVA (A, C), One Sample T and Wilcoxon Test (B, D). Scale bars equal 50um in all confocal images.
